# Piezoelectric Response and Substrate Effect of ZnO Nanowires for Mechanical Energy Harvesting in Internet-of-Things Applications

**DOI:** 10.3390/ma15196767

**Published:** 2022-09-29

**Authors:** Mateusz Wlazło, Maciej Haras, Grzegorz Kołodziej, Oliwia Szawcow, Jakub Ostapko, Wojciech Andrysiewicz, Dzmitry S. Kharytonau, Thomas Skotnicki

**Affiliations:** 1 CBRTP—Research and Development Center of Technology for Industry, Waryńskiego 3A, 00-645 Warsaw, Poland; 2 Centre for Advanced Materials and Technologies CEZAMAT, Warsaw University of Technology, ul. Poleczki 19, 02-822 Warsaw, Poland; 3 CENTERA Laboratories, Institute of High-Pressure Physics, Polish Academy of Sciences, 01-142 Warsaw, Poland; 4 Soft Matter Nanostructures, Jerzy Haber Institute of Catalysis and Surface Chemistry, Polish Academy of Sciences, Niezapominajek 8, 30-239 Krakow, Poland; 5 Institute of Microelectronics and Optoelectronics, Faculty of Electronics and Information Technology, Warsaw University of Technology, ul. Koszykowa 75, 00-662 Warsaw, Poland

**Keywords:** piezoelectricity, energy harvesting, zinc oxide, nanostructures

## Abstract

Recently, an unprecedented growth in the internet of things (IoT) is being observed, which is becoming the main driver for the entire semiconductor industry. Reliable maintenance and servicing of the IoT is becoming challenging, knowing that the IoT nodes outnumber the human population by a factor of seven. Energy harvesting (EH) can overcome those difficulties, delivering the energyautonomous IoT nodes to the market. EH converts natural or waste energies (vibrations, heat losses, air flows, light, etc.) into useful energy. This article explores the performance of ZnO nanowires under mechanical actuation to characterize their piezoelectric performance. ZnO nanowires were fabricated using ALD and a subsequent chemical bath growth. AISI 301 steel was used as a substrate of the EH device to better fit the mechanical requirements for the piezoelectric generator. We determined that a thin layer of another oxide below ZnO provides outstanding adhesion. The samples were submitted under repetitive mechanical stress in order to characterize the output piezovoltage for different conditions. They exhibited a piezoelectric signal which was stable after hundreds of actuations. This shows good promise for the use of our device based on ZnO, an Earth-abundant and non-toxic material, as an alternative to the conventional and popular but harmful and toxic PZT. The designed measurement setup demonstrated that a AISI 301 steel substrate coated with ZnO deposited by ALD and grown in a chemical bath has promising performance as a piezoelectric material. Characterized ZnO samples generate up to 80 nJ of energy during 55 s runs under matched load conditions, which is sufficient to supply a modern IoT node.

## 1. Introduction

**Figure 1:**
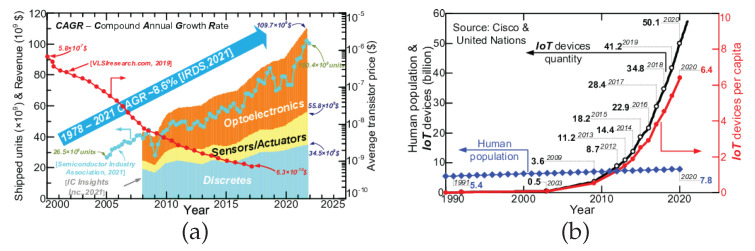
(**a**) History of the semiconductor industry underlining fabrication capability boost [1] (blue line), cost reduction [2] (red line), CAGR [3] (blue arrow), revenue per selected branch [10] (bar plot); (**b**) historical comparison of IoT nodes quantity [5] (black line) with human population [11] (blue line), highlighting the IoT devices per capita (red line).

For more than five decades, the semiconductor industry has continually astonished the market with rising fabrication capacity (delivering more than 100 bn units in 2022) [1] under unprecedented cost effectiveness [2], dumping the average transistor price more than ×1000. This has resulted in an average compound annual growth rate (CAGR) of 8.6% over a 43-year time span [3], a growth rate that has never been seen before (see Figure 1a).

Recently, a new driver for the entire semiconductor industry has appeared, which is the emerging market of the internet of things (IoT) [4]. IoT nodes already outnumber the human population 6.4× and are expected to reach 70 bn units in 2030 [5] (see Figure 1b). The IoT market represents already a branch with a size of ∼$1.6 bn [6,7] and exhibited an outstanding average CAGR of ∼39% over 2015–2021 [8]. Surprisingly, the IoT growth could have been even faster, but it has been impaired by the lack of alternatives for battery and wire supply. The principle of IoT is to establish communication between small electronic devices, called Things, e.g., temperature sensors, presence detectors, and pressure sensors, distributed upon a large area. Communication between Things omits human intervention [9]. Current challenges for IoT include the size, portability, location spots and quantity of devices, and providing power supply for each IoT device node. Conventional supply (batteries or wires) is burdened with important drawbacks. Batteries require periodic checks and replacements if needed. This requires direct access to each of the nodes, and without significant breakthroughs, would soon give full-time jobs for all of humanity. Additionally, batteries have a negative impact on the environment in the form of tons of used-batteries trash. Wire supply is expensive and difficult to modify, which significantly reduces the portability and mobility of Things. Therefore, energy autonomy would be a huge relief for IoT, paving the way to future growth and expansion to the market.

**Figure 2:**
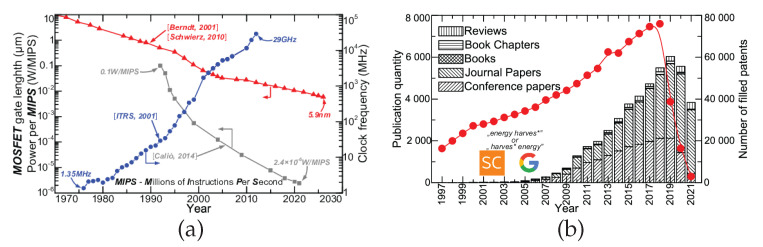
(**a**) Historical evolution of the electron devices performance revealing the miniaturization trend [13,14] (red line), operational frequency boost [15] (blue line) and reduction of power required per millions instruction per second (MIPS) [16] (gray line); (**b**) development of the energy harvesting in number of scientific publications after Scopus (bar plot) and patent applications (red line) following Google Patents.

The fabrication capabilities boost, and cost reduction illustrated in Figure 1a is accompanied by the galloping miniaturization (transistor’s gate length reduced by factor 200×) and performance boost (clock frequency increased 21,000×) (see Figure 2a). Owing to this, modern electronic devices are requiring less and less energy to operate. Over the last three decades, the energy consumption has decreased by a factor of almost 42,000 for the same load.

These two industrial circumstances, IoT market growth and the development of less energy-demanding devices, are driving the research toward alternative power supplies, enabling the elimination of wires and/or batteries. The development of micro- and nano-technologies has opened new possibilities to produce sufficient energy from wastes or naturally available energy sources. This approach defines the branch known as energy harvesting (EH) [12]. EH is nowadays gaining enormous attention from both industrials and academics, which is manifested by the number of filled patent applications and scientific publications (see Figure 2b). A decrease in publications and patents after 2019 can be observed, which can be attributed to the pandemic of COVID-19 that confronted industrial and academic sectors with huge difficulties: cutting costs, production stoppages, temporary lab closure, remote work, confinements, etc. This effect is visible particularly in the share of conference papers which diminished greatly in 2020 and 2021. Nevertheless, the raising trend from before 2019 is expected to be reconstituted soon when the pandemic situation no longer affects industrial and academic working conditions.

**Figure 3:**
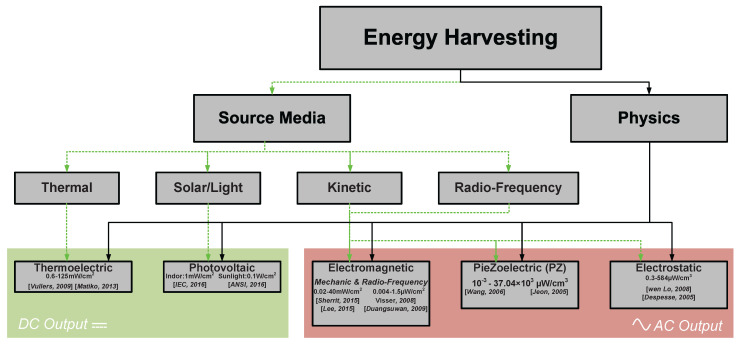
Taxonomy of the energy harvesting techniques with respect to the physical effect and input energy [17,18], highlighting common power densities for domestic environment reported in the literature; thermoelectric [19,20], photovoltaic [21,22], electromagnetic [23,24] (mechanical), [25,26] (radio-frequency), and electrostatic [27,28].

Figure 3 summarizes decades of research and thousands of scientific publications highlighting main conventional EH techniques and illustrating the physics and input energy source.

Commenting on Figure 3, it can be underlined that different energy sources require an adapted EH technique. Different physical effects and topologies are capable of producing different amounts of power. Confronting power densities in the range of μW or mW with power needed to supply modern electron devices (see Figure 2a gray line) or modern IoT nodes requiring ∼10–100 μJ/cycle of operation [18,29,30], it can be concluded that a well-selected EH generator can fully cover the supply requirements of a modern IoT node, offering energy-autonomous devices.

This paper focuses on one of the most common EH techniques, the piezoelectric (PZ). A more focused literature review of PZ harvesters is provided in Table 1.

PZ converts mechanical energy (vibrations, stress) into electric voltage, owing to the piezoelectric effect discovered in 1880 by Jacques and Pierre Curie [31]. This effect was observed in crystals, such as tourmaline, topaz, calamine, and quartz, but not in amorphous materials. The first application of this effect was the ultrasonic submarine detector constructed in France in 1917 [32].

Since their discovery, huge improvements in PZ materials have been obtained. This market of piezoelectric devices was estimated at USD14.3 billion in 2010. The use of PZ materials is very wide ranging, from well-known high voltage generators in lighters to infertility treatment [33]. Piezoelectrics are mainly used as sound sensors [34], as cantilevers in atomic force microscopes [35], noise and vibrations attenuators [36], motors [37], and, recently, as energy harvesters [19,38,39,40]. The piezoelectric effect is reversible. A mechanical deformation can be achieved by applying an electric potential to the material [40]. The reverse piezoelectric effect is very useful in various diagnostic applications, e.g., wrist blood pressure or heart beat rate monitoring [41]. It is also used to detect fatigue cracks in materials [42], e.g., wind turbines rotor blades [43].

PZ offers single-step conversion by directly transforming mechanical vibrations into electric potential. The material is therefore the crucial point of this conversion. The most popular piezoelectric material is the lead zirconium titanate (PZT) [44,45,46,47,48,49,50,51,52,53,54,55,56,57,58]. Its high cost and lead (Pb) content have motivated research on alternative materials. A widely studied example is zinc oxide, which is composed of abundant and non-toxic elements. Of particular note is the possible synthesis in the form of unidirectional nanowires [59,60,61,62,63] which enhances the piezoelectric effect in the preferred axis. The directionality of the nanowires highly depends on the substrate on which they are grown, which is also an important issue concerning the current results described further in the text.

In recent years, focused research effort has been directed toward components fabricated on flexible substrates [58,64,65,66] that greatly expand the possible range of applications served by EH components. The current research is in line with this trend.

**Table 1:** Summary of reported piezoelectric harvesters and their parameters.

f	Excitation	Mass	Volume	P	Power Density	Material	Reference
(Hz)	(m/$s^{2}$)	(g)	(cm${}^{3}$)	(μW)	(μW/cm${}^{3}$)		
0.5	N/A	1.2	0.101	0.25	2.47	PZT-5H	[52]
1.1	N/A	N/A	25	8400	336	PZT	[44]
100	72.7	0.96	0.20	35.5	16.3	PZT	[48]
120	2.5	9.2	1	375	375	PZT	[46]
120	0.98	N/A	N/A	500	N/A	PMNZT	[67]
13.9	106	N/A	27·10${}^{-6}$	1	37.04·10${}^{3}$	PZT	[49]
1500	3.92	9·10${}^{-4}$	0.005	0.03	60	AlN	[68]
N/A	N/A	N/A	8.19·10${}^{-4}$	9.24·10${}^{-6}$	11.28	AlN	[65]
230	9.8	N/A	N/A	0.27	N/A	PZT	[55]
40	2.5	52.2	4.8	1700	700	PZT	[50]
462.5	19.6	N/A	N/A	2.15	N/A	PZT	[56]
50	N/A	N/A	9	180	20	PZT	[51]
56	N/A	228	113	1·10${}^{5}$	2650	P1-89 PZT	[54]
608	9.8	0.0016	0.0006	2.16	3600	PZT	[53]
67	4	2.8	0.987	240	243.1	PZT	[57]
7000	N/A	N/A	N/A	1600	N/A	PZT-PIC255	[47]
80	2.3	0.8	0.128	2.1	16.4	PZT	[45]
1	N/A	N/A	0.04	5.6	140	PZT/PVDF	[58]
3	N/A	N/A	N/A	1.296·10${}^{-6}$	2.47	PVDF micro wall	[64]
N/A	N/A	N/A	0.045	1.03	22.8	ZnO NWs	[63]
N/A	N/A	N/A	0.13·10${}^{-3}$	0.1·10${}^{-3}$	1.28	ZnO NWs on paper	[61]
N/A	N/A	N/A	N/A	N/A	1·10${}^{-3}$	ZnO NWs	[59]
N/A	N/A	N/A	N/A	N/A	10	ZnO NWs	[60]
3	N/A	N/A	N/A	N/A	144	ZnO NWs	[62]
1.63	N/A	N/A	4.51·10${}^{-4}$	1.38·10${}^{-3}$	3.1	ZnO NWs on steel	(this work)

PZT—Lead zirconium titanate, Pb[Zr${}_{x}$Ti${}_{1-x}$]O${}_{3}$, PMNZT—PZT co-doped with Mg and Nb, ZnO NWs—zinc oxide nanowires, PVDF—polyvinylidene difluoride, N/A—data not available.

## 2. Materials and Methods

### 2.1. Sample Fabrication

#### 2.1.1. Substrate

For piezoelectric material deposition, austenitic–ferritic stainless steel (EN 1.4310/AISI 301 class) was chosen as the substrate due to its particular mechanical properties. This type of steel exhibits high yield strength and retention of shape after prolonged application of strain. The raw material in the form of 100 μm-thick sheets was cut into rectangular 20 × 50 mm samples. The substrate itself acted as the bottom electrode. It should be noted that a device with optimized substrate resistivity may have offered better PZ efficiency at the cost of inferior mechanical properties.

The AISI 301 steel is a novel type of substrate. For the verification of the growth conditions, selected processes were performed additionally on polished silicon (Si) substrates. If not otherwise explicitly indicated, we will refer to samples deposited on the steel substrate.

#### 2.1.2. Synthesis

**Figure 4:**
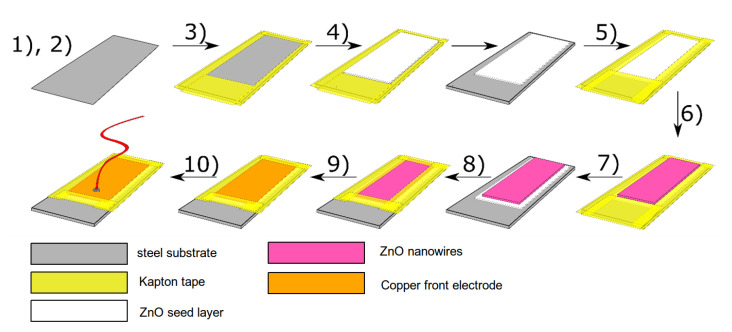
Manufacturing of the sample. (**1**) Cutting substrate (steel) into desired dimensions. (**2**) Degreasing and chemical cleaning of the substrate. (**3**) Masking of the substrate with Kapton tape prior to ALD deposition. (**4**) Deposition of adhesion layer and seeding layer via ALD on metal substrate. Materials employed were Al${}_{2}$O${}_{3}$ and ZnO. (**5**) Masking of the substrate with Kapton tape prior to hydrothermal growth. (**6**) Hydrothermal growth of ZnO NWs via hydrothermal method on exposed seeded surface. (**7**) Cleaning from unwanted residues. (**8**) Masking of the sample with Kapton tape before mounting of top electrode. (**9**) Placing and immobilization of top copper electrode to achieve contact only with Kapton tape and surface of ZnO nanowires. (**10**) Wiring of the top electrode.

The following textual description of synthesis steps is illustrated in Figure 4 in graphical form. After cutting to the desired dimensions, the steel substrates were masked using Kapton tape resulting in a 18 × 35 mm exposed surface. Then, the ALD layers were deposited. The role of ALD deposition was to enable the growth of piezoelectric ZnO nanowires (NWs) while ensuring good adhesion to the bottom electrode surface. ALD deposition was performed using Beneq P-400 semi-industrial reactor with increased cost-effectiveness, allowing for hundreds of substrates throughput per process. The reactor was kept heated to 200 °C throughout the processes. Diethylzinc (DEZ) [Lanxess, CAS 557-20-0, purity ≥ 99.5% m/m], trimethylaluminum (TMA) [Lanxess, CAS 75-24-1, purity ≥ 99.8% m/m] and deionized water (H${}_{2}$O) were used as zinc, aluminum and oxygen precursors. The reagents were kept at room temperature with the exception of H${}_{2}$O, which was heated to a constant temperature of 28 °C. Nitrogen gas (99.99% N${}_{2}$) with a flow rate of 2500 sccm (square cubic centimeter per minute) was used as a carrier and purge gas. Aluminum oxide (Al${}_{2}$O${}_{3}$) films were obtained by sequential dosing of H${}_{2}$O for 350 ms and TMA for 300 ms. The pulses were separated by 3 s purges of N${}_{2}$. This cycle was repeated a set number of times to obtain a desired thickness (assuming thickness growth per cycle GPC${}_{Al2O3}$ = 0.1 nm). Zinc oxide (ZnO) films were deposited in a similar manner by dosing H${}_{2}$O for 350 ms and DEZ for 300 ms with a 3 s purge of N${}_{2}$ between precursor pulses (GPC${}_{ZnO}$ = 0.167 nm). At the end of each process, a 600 ms pulse of H${}_{2}$O was delivered to ensure full oxygen saturation, followed by a 10 s N${}_{2}$ purge.

Chemical bath deposition (CBD) was performed by placing the samples on which ALD layers were deposited in an aqueous solution of zinc nitrate hexahydrate Zn(NO${}_{3}$)${}_{2}$·6H${}_{2}$O [Chempur, Karlsruhe, Germany] and hexamethylenetetramine (HMT, C${}_{6}$H${}_{12}$N${}_{4}$) [Thermo Scientific, Waltham, MA, USA]. The solution concentration varied between 30 and 100 mM. The molar ratio of the reagents was kept constant at 1:1, replicating the conditions described in Refs. [69,70]. For each of the processes, the samples were left in the solution for 4 h. The reaction containers were placed on a heated surface—a hotplate or a magnetic stirrer, in both cases set to 95 °C. After the deposition process the samples were rinsed with DI water for 5 min in an ultrasonic bath.

Finally, the samples were once again masked with Kapton tape, resulting in a 16 × 32 mm exposed surface that was put into contact with a copper electrode which acted as the top electrode.

### 2.2. Characterization

#### 2.2.1. X-ray Diffraction

The crystal structure of the formed ZnO nanowires was examined by the X-ray diffraction (XRD) technique using an X’Pert PRO PANalytical B.V diffractometer. XRD patterns were collected in the 2θ range of 10–100° using Ni-filtered Cu Kα radiation operating at 40 kV and 30 mA.

#### 2.2.2. SEM

Scanning electron microscopy (SEM) was performed using the Thermo Fisher Phenom Pharos system. The system is equipped with an energy-dispersive X-ray spectroscopy (EDS) attachment, which was used to confirm the presence of expected layers by analyzing the Zn and O content on the surface. The applied electron beam acceleration voltage was 12–15 kV (imaging mode) or 5 kV (EDS mode).

**Figure 5:**
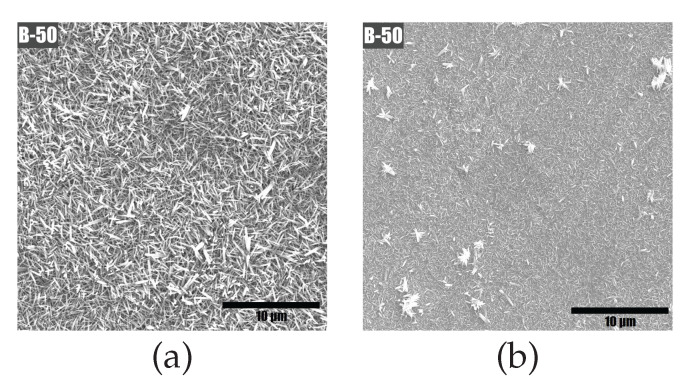
Scanning electron top-view micrographs of sample B-50 showing the ZnO nanowires on samples coming from a process carried out in a container vessel placed on a hotplate (**a**) and on a heated magnetic stirrer (**b**).

**Figure 6:**
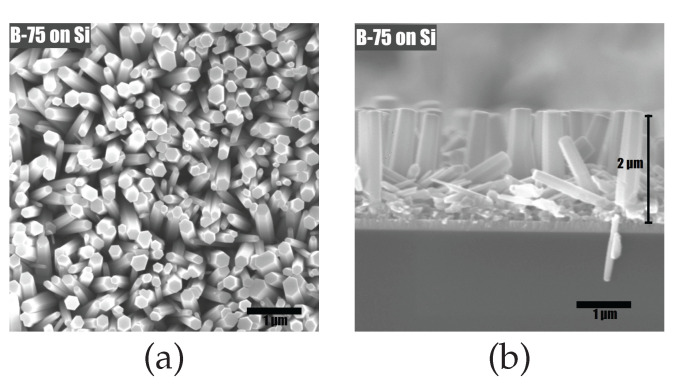
Scanning electron (**a**) top-view and (**b**) cross-section micrographs of sample B-75 deposited on silicon.

The morphology of NWs produced by using the hotplate was found to be more uniform than when using the stirrer. There was a difference in the amount of scattered aggregates, whose dimensions were much larger than the NWs themselves (see Figure 5). The resulting inhomogeneity is generally believed to be detrimental to the piezoelectrical properties of the sample. Conversely, a continuous coating of uniformly shaped and highly oriented structures is seen as desirable [71]. Such a uniform and oriented coating was obtained on the silicon substrates used to verify the growth conditions (see Figure 6).

#### 2.2.3. Piezoelectric Effect Measurements

**Figure 7:**
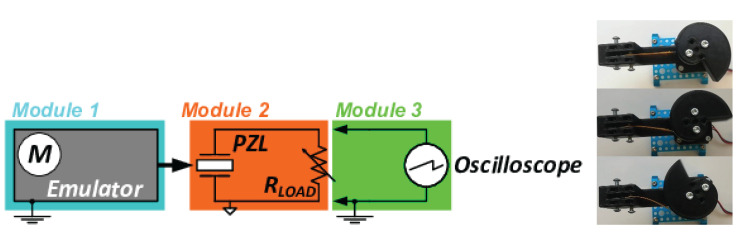
Electrical diagram of the test bench utilized for the evaluation of the piezoelectric response of ZnO samples under repeated mechanical deformation (**left**) and snapshots showing the action performed by the spiral actuator (**right**).

The first module (leftmost in Figure 7), called the emulator, consisted of an actuator which applied mechanical strain to the sample. The actuator was designed in the shape that can be approximated by the logarithmic spiral equation r = aexpkϕ (with *a* > 0), starting at ϕ = 0 and ending at ϕ = 2nπ (n = 1, 2, …). The spiral actuator manufactured by 3D printing (ABS filament) was mounted horizontally on the shaft of a step motor (Makeblock 42BYG) through a hole in the middle in such a manner that the rotation of the shaft resulted in the rotation of the actuator. The step motor was controlled by a single-board computer (Raspberry Pi) running Raspbian OS.

The role of the second module was to provide a mounting point for the sample (middle of Figure 7). Rectangular samples were placed inside a clamp by one of its shorter sides. The clamp had electrical contact only with the bottom electrode (steel substrate). To facilitate electrical contact to the sample, the bottom electrode was additionally tightly screwed to the clamp. An electrode made from a sheet of copper cut to the dimensions of the sample and attached on top was wired directly to the measurement system. The clamp itself was placed on a rectangular ABS part that acted as an insulating table that eliminated possible contact between the metal clamp and frame.

The two modules were attached to a shared construction frame. The distance between the actuator and the sample could be adjusted without disconnecting the sample. This allowed performing measurements on samples with different dimensions. In addition, changing the distance can aid in adjusting the intensity of mechanical actuation delivered to the sample.

The rotation of the spiral shape allowed to deliver two kinds of strain to the sample. In the first mode, the sample is bent and unbent at an angle and speed controlled in a precise manner by step motor programming and the adjustable distance to the sample. In the second mode, the spiral is rotated beyond the endpoint, which results in the sample being gradually bent and then rapidly released.

The third module was the measurement and data acquisition system (rightmost in Figure 7). Its main hub was a Keysight DSOX2024A oscilloscope connected in parallel to the second module through a variable resistive load realized by a resistance box. During one measurement cycle, the load was first set to the maximum available load R${}_{MAX}$ = 1.111111$\cdot 10^{7}\;\Omega$ and then gradually reduced by turning one knob of the resistance box at a time down to R${}_{MIN}$ = 2.111$\cdot 10^{4}\;\Omega$. The values of R${}_{MAX}$ and R${}_{MIN}$ were chosen based on the expected impedance of the sample between R${}_{LOAD}$ = 10${}^{6}$–10${}^{7}$ Ω.

**Figure 8:**
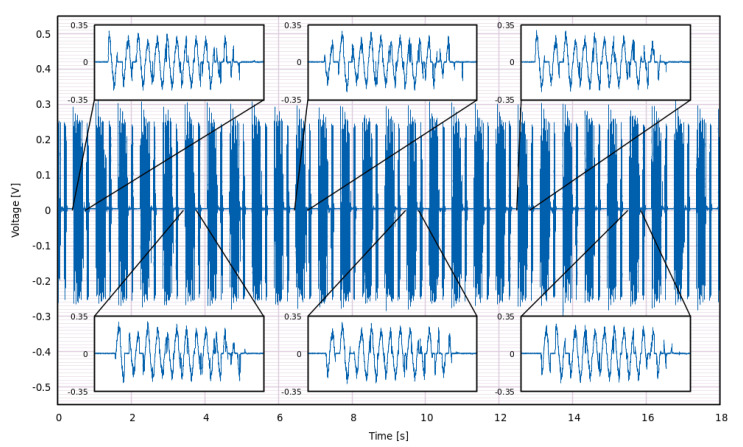
Voltage waveform generated by sample B-75 under R${}_{LOAD}$ = 6.11 MΩ upon repeated actuation under test conditions. Insets show waveforms of single subsequent actuations. The length of a single displayed waveform is 0.5 s and the start points between subsequent actuations are separated by 0.61 s.

The data were saved in the form of voltage waveform vs. time V(t) for further analysis. A fragment of one of the typical waveforms generated by our samples when displaced by the emulator module is displayed in Figure 8. It was recorded at optimal external load, i.e., the one under which the calculated generated energy was the highest.

## 3. Results and Discussion

The ALD and CBD processes were performed on samples including the full size AISI 301 steel substrates, as well as on smaller pieces which were used for SEM imaging. The processes listed in Table 2 were carried out with heating provided by a hotplate. Two types of ALD layers were used as seed layers. The first type, labeled **A**, comprised ∼200 nm ZnO layers.

**Figure 9:**
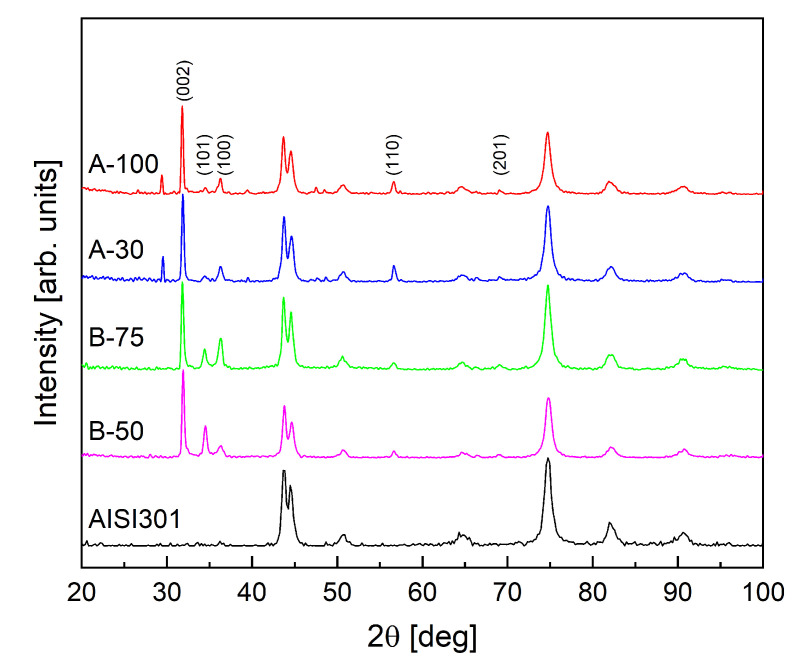
XRD patterns of examined ZnO structures. Data for AISI 301 substrate is shown as a reference.

**Figure 10:**
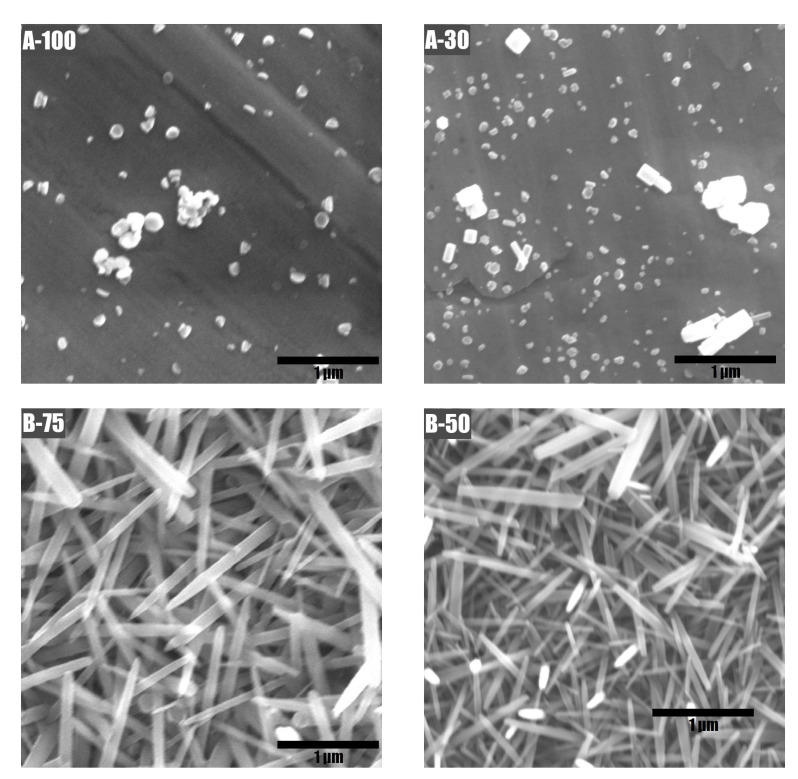
Scanning electron top-view micrographs of sample A-100 (top left), A-30 (top right), B-75 (bottom left), B-50 (bottom right).

The results of the XRD analysis are presented in Figure 9. XRD patterns of all obtained materials show well-defined diffraction peaks of the crystalline ZnO phase with hexagonal wurtzite structure (JCPDS card # 36-1451) [72]. In the case of the A-100 and A-30 samples, recorded XRD patterns have an additional diffraction peak at 2θ of 29.55° of relatively high intensity. Its occurrence may indicate the presence of microstrains in the samples, which are usually the results of crystal imperfection and distortion. On the other hand, this additional peak may indicate the presence of phase impurity in these samples [73,74]. This is also supported by SEM images of these layers (Figure 10), showing two different morphologies of ZnO structures formed on the surface. In turn, XRD patterns of the B-75 and B-50 samples show peaks corresponding to pure wurtzite structure. Moreover, XRD patterns of these samples have a much more pronounced relative intensity of the diffraction peak at 2θ of ca. 34.50°, corresponding to the (002) plane. This indicates that the growth orientation of the formed ZnO nanowires in this case is along the c-axis of the hexagonal crystals [75]. The diffractogram of the B-50 sample showed the broadest (002) peak, corresponding to the formation of ZnO structures with the smallest crystallite size. The crystallite size (D) of the formed ZnO structures was calculated using the Debye–Scherrer equation:

$$D=\frac{0.9\lambda }{\beta \cos\theta },$$

where *D* is the mean size of the crystallite, 0.9 is the dimensionless shape factor, λ is the X-ray wavelength, β is the line broadening at half of the maximum intensity, and θ is the Bragg angle. The obtained parameters for the (100) and (002) planes are summarized in Table 3.

The calculated values for the A-100, A-30, and B-75 samples are almost identical, suggesting good reproducibility and versatility of the used technique. The B-50 sample has the smallest value of crystallite size among the examined samples. The presence of such nanocrystallites significantly increases the surface area in electrical measurements.

Figure 10 shows magnified images of each of the four samples (A-100, A-30, B-75, and B-50) which display the different growth mode depending on the type of ALD layer. It reveals that the growth of nanowires on the type **A** seed layer was minimal and scattered in very few randomly distributed sites on the surface. On the other hand, the ALD layer labeled **B** provided adequate conditions for the uniform growth of the nanowires.

SEM images were used to calculate the volume of the active layer, which was then used to estimate the generated power density in the piezoelectric effect generation measurements. ZnO grown in the form of nanowires features considerable space gaps between each individual nanowire. Using the cross-section image presented in Figure 6b, the typical length of a single nanowire was estimated to be ∼2 μm, amounting, together with the seed layer of 200 nm, to the total possible thickness of ∼2.2 μm.

As evidenced by SEM images (e.g., in Figure 5a), in contrast to the crystalline silicon substrate, ZnO nanowires on AISI 301 substrate grow in a more disoriented manner. The nanowires appear to grow at an angle to the surface. This is attributed to the lack of a uniform crystal structure of the substrate. Similar to the Si substrate, there is a considerable amount of gap space between the wires. Taken these aspects into account, the filling factor of the active layer was estimated at 40% for power density calculations. Thus, the volume of the piezoactive layer used for power density calculations was equal to 16 mm × 32 mm × 2 μm × 0.4 = 4.51·10${}^{-4}$ cm${}^{3}$.

**Figure 11:**
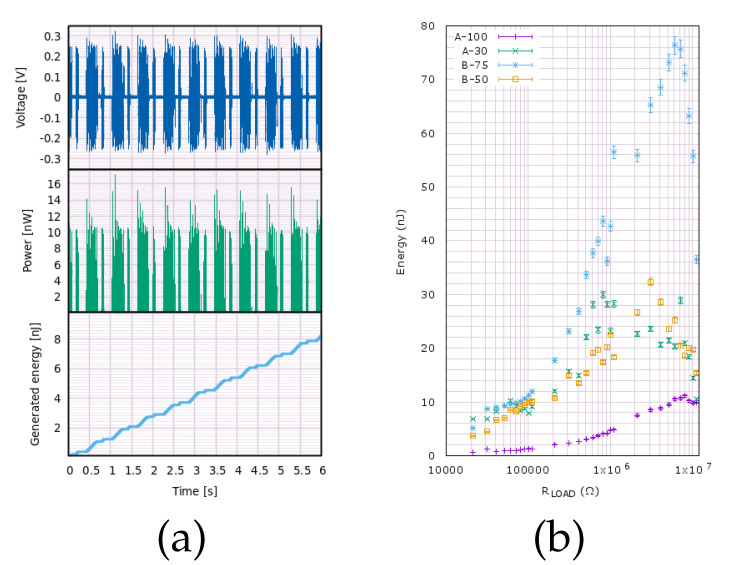
(**a**) Recorded waveform V(t) (top), instantaneous power $P\left(t\right)=V^{2}\left(t\right)/R_{LOAD}$ (middle) and accumulated energy calculated by numerically integrating the signal $E\left(T\right)=\int_{0}^{T}P\left(t\right)dt=\int_{0}^{T}V^{2}\left(t\right)dt/R_{LOAD}$ (bottom) for sample B-75; (**b**) energy accumulated in a single production run of 90 actuations vs. resistive load Please provide scale for figure, if any.

Using the voltage recorded for each sample, instantaneous power was computed according to Ohm’s law, $P\left(t\right)=V^{2}\left(t\right)/R_{LOAD}$, where R${}_{LOAD}$ is the resistive load set on the resistance box. Then the value of energy accumulated from the beginning of the measurement up to t = T was computed by numerical integration of power with respect to time, $E\left(T\right)=\int_{0}^{T}P\left(t\right)dt=\int_{0}^{T}V^{2}\left(t\right)dt/R_{LOAD}$ (see Figure 11a).

Recorded waveforms included the signal generated by piezoelectric samples as well as a certain amount of noise. Both of these types of waveforms were integrated when computing the generated energy. For higher values of R${}_{LOAD}$, the contribution of noise was negligible. However, when lower values of R${}_{LOAD}$ were used, even the low voltage values contributed significantly to the integrated energy. This was alleviated by introducing a cutoff value V${}_{CUTOFF}$. Below this value, the recorded voltages were ignored as if V(T) was equal to 0. Cutoff values between 0 and 5 mV were tested. It was found that V${}_{CUTOFF}$ = 5 mV was the appropriate setting, taking into account that the undesired influence of noise at low R${}_{LOAD}$ values was mitigated while keeping energies computed at high R${}_{LOAD}$ essentially unchanged. The high signal-to-noise ratio shows that the samples and experimental setup were well prepared.

Waveforms were recorded at each R${}_{LOAD}$ value for a total of 55.2 s. During this period, the samples were displaced 90 times by the emulator, resulting in actuation frequency equal to 1.63 Hz. This procedure was repeated for 30 different values of R${}_{LOAD}$, amounting to a total of 2700 mechanical actuations delivered to each sample. Across the measurements, the voltage signal was stable.

The amount of accumulated energy is displayed in Figure 11b as a function of the resistive load connected to each sample. Energy generation parameters are listed also in detail in Table 4. For each of the measured samples, a maximum value of accumulated energy was obtained at a certain resistive load R${}_{LOAD}$ value. This is the value in which the internal impedance of the sample is matched to the external impedance of R${}_{LOAD}$. Depending on the sample, the matched impedance value was between 0.81 and 8.11 MΩ. It should be noted, however, that the energy-load profile is the most spread out for the sample that appears to have the lowest impedance, A-30. The power was just 0.02 nW lower at R${}_{LOAD}$ = 7.11 MΩ. This places it much closer to impedance-matched conditions measured for other samples. Other samples, in particular type-**B** have a more clearly defined maxima. This leads us to believe that for these two samples, the highest energy points indeed correspond to the impedance-matched conditions.

The highest amount of energy is generated by sample B-75—76.37 nJ—at the optimal setting of the resistive box. This corresponds to the amount of power equal to 1.38 nW. Taking into account the volume of the active layer, the power density can be calculated at 3.1 μW/cm${}^{3}$. Compared to other reported piezoelectric harvesters (see Table 1), this value places it in the middle of reported power densities for harvesters based on ZnO nanowires. It is slightly higher but comparable to ZnO synthesized on a paper substrate, which is also a flexible substrate, similar to deposition on the AISI 301 steel reported here, and may serve similar applications in mechanical energy harvesting.

Devices which draw few nanowatt sleep power and 16 pJ per transmitted bit are realized in the research of wireless sensors [52]. These could be powered easily by a small array of piezoelectric devices described here. The growth methods also allow for the deposition of a thicker layer (ALD) or vertically stacked structures [76]. Both of these options might be engineered to contribute to an increased piezovoltage in future applications.

### Error Analysis

The characterization of 2D materials is challenging because of the big impact of leakages and parasitic effects introduced by the measurement circuit. Therefore, in order to minimize the impact of the measurement circuit on the characterized ZnO nano-rods piezoresponse, large-surface metallic electrodes were used. Additionally, the access electrodes are fixed using screws, which provide small contact resistivity under significant mechanical vibrations. Figure 7 presents the characterization circuit used during this experiment. It can be observed that the ZnO nanowires piezovoltage is measured by the oscilloscope. According to the technical documentation of the oscilloscope [77], the maximal error along the Y-axis (voltage error) induced by the oscilloscope is 4.25%. In order to minimize the impact of this error, a statistical data treatment upon the population of at least three measurements was implemented.

## 4. Conclusions

In this work, we demonstrated the growth of ZnO nanowires from a chemical bath on a novel type of substrate—AISI 301 steel. The substrate was chosen with mechanical energy harvesting applications in mind. After its deposition, the measured voltage upon repeated mechanical actuation provided the means of verifying its applicability in internet-of-things applications.

The nanostructured active layer was deposited directly on the substrate, which was first prepared by seeding process using atomic layer deposition of thin oxides. A typical procedure in which pure zinc oxide is used as a seed layer to catalyze the nanowire growth was modified to account for the particular surface properties of the AISI 301 substrate. The seed layer modification, by inserting a 20 nm-thick layer of another oxide Al${}_{2}$O${}_{3}$, was found to eliminate the adhesion issues of the ZnO ALD layer.

The measured voltage signal was stable upon thousands of actuations of each sample, confirming the resilience of the piezo layer to sustained mechanical stress. Moreover, the piezoactive layer generated a considerable amount of power, enough for typical operations performed by IoT devices. The power density was comparable to other reported values (see Table 1). In particular, it was on the same order of magnitude as the ZnO nanowires fabricated on another type of flexible substrate. These two factors, when combined, show high promise for the final application of the device as a part of a power generation component for a IoT wireless sensor node.

The presented fabrication technology of the ZnO nanorods is economically attractive and can be easily upscaled. Moreover, the presented ZnO nanogenerators exhibit an interesting piezoresponse which can be boosted using topological and material optimization. Additionally, the characterization of the harvesting performance in the function of frequency will add important information of how to mechanically tune the harvester to fixed vibrations in order to reach the optimal output energy.

Beyond its technological and economical attractiveness, the ZnO nanowire fabrication technology can be easily upscaled, enabling NW fabrication upon very large surfaces. This particularity, besides the high harvested output power, enables countless possibilities in sensing applications. A characterization of the inverse piezoelectric effect (excitation of vibrations when applying an AC voltage) in the presented ZnO nanorods is an inspiring and interesting research path. Such research will enable the cheap and large-scale production of crack detectors in long and big objects, such as airplane wings or train rails.

## Figures and Tables

**Table 2:** Growth parameters used in ALD and CBD processes of samples’ fabrication.

Sample ID	ALD Seed Layer	Reaction Solution Concentration (mM/dm${}^{3}$)
A-100	200 nm ZnO (**A**)	100
A-30	200 nm ZnO (**A**)	30
B-75	20 nm Al${}_{2}$O${}_{3}$/200 nm ZnO (**B**)	75
B-50	20 nm Al${}_{2}$O${}_{3}$/200 nm ZnO (**B**)	50

**Table 3:** Position of main XRD peaks and calculated crystallite size of examined ZnO structures.

Sample ID	2θ [deg]		D [nm]	
	(100)	(002)	(100)	(002)
A-100	31.79	34.48	27.48	26.42
A-30	31.86	34.43	27.49	26.43
B-75	31.81	34.42	27.48	26.43
B-50	31.89	34.51	20.61	19.82

**Table 4:** Piezoelectric energy generation parameters obtained from measurements of voltage generated by the samples.

Sample ID	Optimal Impedance	Max. Accumulated Energy	Max. Power	Max. Accumulated Energy Density	Max. Power Density
	MΩ	nJ	nW	μJ/cm${}^{3}$	μW/cm${}^{3}$
A-100	0.81	29.94	0.54	66.5	1.2
A-30	8.11	11.18	0.20	24.8	0.4
B-75	6.11	76.37	1.38	169.5	3.1
B-50	3.11	32.36	0.58	71.8	1.3

## Data Availability

Data available from the authors at request.
